# Psychological Demands of International Rugby Sevens and Well-Being Needs of Elite South African Players

**DOI:** 10.3389/fpsyg.2019.00676

**Published:** 2019-03-29

**Authors:** Ninette Kruyt, Heinrich Grobbelaar

**Affiliations:** Department of Sport Science, Faculty of Medicine and Health Sciences, Stellenbosch University, Stellenbosch, South Africa

**Keywords:** rugby sevens, performance, team culture, psychological skills, mental strategies, career termination, mental (ill) health, well-being

## Abstract

Rugby sevens was included in the 2016 Olympic Games, with South Africa’s Blitzboks winning bronze. They also won the 2016/2017 and 2017/2018 World Rugby Sevens Series. Whilst peak performance is paramount at the elite level there is a growing responsibility to address player well-being and off-the-field player needs. This study explored the psychological demands of international rugby sevens and the well-being needs of elite players. Twenty professional players (age range: 21–33 years) participated in semi-structured interviews. Qualitative content analysis yielded five categories of psychological demands: (1) tournament structure, (2) resilience, (3) cope with physicality, (4) perform when fatigued, and (5) perform under pressure. The prevailing team culture included: (1) team cohesion, (2) clear purpose, (3) work ethic, (4) team values, (5) happy environment, (6) relationships with coaching staff, and (7) faith. Various psychological skills [(1) goal-directed behavior, (2) compartmentalization, (3) deal with anxiety, (4) motivation, (5) imagery, and (6) self-confidence] and mental strategies [(1) coping, (2) “back-to-zero,” (3) creativity, and (4) cognitive triggers] utilized by the players are discussed. Their general well-being and individual needs were: (1) physical needs, (2) financial concerns/needs, (3) preparation for life after rugby career, (4) support structures, and (5) mental (ill) health. This information could be useful to develop an integrated sport psychological and well-being program aimed at improving performance and facilitating psychological well-being both during and after retirement from elite sport.

## Introduction

Globally, the popularity of rugby sevens has increased, partly due to its inclusion in the 2016 Olympic Games (Higham et al., 2012; Lopez et al., 2012). This event has been a catalyst for research into this sport (Tucker, 2016). Higham et al. (2013) noted that players will begin to specialize in either fifteens or sevens. Due to its inclusion in the Olympics many 15-a-side players attempted the switch to sevens to take part in this global spectacle.

Sevens is played on the same field and has similar rules to fifteens (Higham et al., 2012, 2013). The main differences are fewer players (seven players on the field and five reserves), shorter matches (two seven-minute halves) and multiple matches during tournaments (Higham et al., 2012; Lopez et al., 2012; Suarez-Arrones et al., 2012). International 15-a-side (test) matches are typically separated by a week, allowing teams to recover and travel, adapt to the new conditions and prepare for the upcoming match. The annual HSBC World Rugby Sevens Series, consists of 10 tournaments over a six-month period, split into five continental tours, each consisting of two tournaments in different countries over consecutive weekends. Tournaments take place over a two or three-day period (World Rugby, 2016). Teams typically play three group stage matches on day one followed by one to three play-off matches on day two, with a waiting period of around 3 h between matches. Players need to adapt to traveling, different climates and time zones, as well as the frequency of games (Van Rooyen, 2015).

Sevens is played at a higher intensity than fifteens (Higham et al., 2012, 2013), with frequent accelerations and incomplete stops contributing to the accumulation of fatigue (Carreras et al., 2013). Sevens players should possess similar or superior physical abilities (e.g., acceleration, speed, power, aerobic and anaerobic capacity) to 15-a-side players (Higham et al., 2013). The body composition of sevens players are similar to 15-a-side backline players, with lower fat percentages enabling greater speed and anaerobic endurance. Teams tend to have multiple high intensity training sessions per day that consist of high running loads, frequent changes in speed and repeated maximal sprint efforts (Higham et al., 2012, 2013; Carreras et al., 2013). This has implications for the preparation of players switching between rugby formats.

Peak physical conditioning will allow players to recover faster between matches, from day one to two and from one tournament to the next (Higham et al., 2013). Injuries sustained during tournaments increase the workload of the remaining players making them more prone to experience fatigue and subsequently injuries (Van Rooyen, 2015). Recovery strategies is needed to reduce fatigue and ensure consistent performance over consecutive days (Higham et al., 2012, 2013).

Whilst mental preparation is key to sevens success (Van Rooyen, 2015), there appears to be a paucity of information on psychological factors in sevens. Osborne Finekaso and Treharne (2019) recently explored sources of stress (training camp, competition and organizational stressors) experienced by professional Fijian rugby sevens players and the detrimental effects thereof. Furthermore, they identified a broad range of coping strategies utilized by these players in these different settings to ameliorate these stressors. Psychological development is often neglected in talent development programs (MacNamara et al., 2010a). Psychological factors should be included in talent development programs as it influence the pathways players follow to reach the elite level (MacNamara et al., 2010b). Psychological skills become a determining factor of success when players possess similar skill levels and physical attributes (Hendricks, 2012). Competitiveness, commitment and self-belief contribute to the on-field performance of players and the development and use of these attributes should be a priority from an early age (MacNamara et al., 2010a).

To develop a psychological skills intervention for a specific sport or athlete the physical, technical, tactical, logistical and psychological demands, and individual player needs should be considered (Taylor, 1995). In light of this statement, the aim of the study was to explore the perceived psychological demands of the sport and off-the-field needs of professional rugby sevens players. In-depth and context rich information will be gained that could guide holistic player development within the existing talent development structures.

## Materials and Methods

The study was exploratory in nature, and utilized a purposive sample and qualitative approach to analyze participants’ subjective views. This allows for a better understanding of the perspectives and experiences of participants (Hiatt, 1986).

Permission was granted by the South African Rugby Union’s Internal Research Review Committee and the Research Ethics Committee of Stellenbosch University (SU-HSD-002671). Participation was voluntary and informed consent forms were signed in accordance with the Declaration of Helsinki.

Twenty of the 27 contracted South African rugby sevens players for the 2016/2017 season took part in the study, with their ages ranging from 21 to 33 years. The sample included nine Olympians, eight national sevens team players (Blitzboks) and three national sevens academy players. Seventeen participants previously represented the national team in the World Rugby Sevens Series ranging from two to 68 tournaments.

A semi-structured interview script was developed that consisted of open-ended questions relating to the perceived physical and psychological demands of international sevens and the off-the-field needs of elite players. NK first interviewed five coaching staff members, including the team’s sport psychologist to refine the interview script. The final data analysis did not consider this information. Before interviewing commenced the participants were informed about the study’s objectives and procedures, that they could withdraw at any time and without prejudice and that they did not have to answer all the questions. The interviews took place in a familiar room at the team’s training facility and following a familiarization period with the interviewer, aimed at building rapport to enhance the trustworthiness of the data. NK conducted all the interviews with the players who were able to construct and voice their own experiences. The semi-structured nature of the interviews enabled probing for additional information from the participants to explore their experiences comprehensively. The interviews were recorded and lasted 40 min on average. All interviews were transcribed verbatim and the information was treated confidentially and reported anonymously.

## Data Analysis

The interview transcripts were analyzed using qualitative content analysis. Data analysis commenced during the data collection phase already, by reading and rereading the scripts, in order to become familiar with the text (Braun and Clarke, 2006). Both authors individually extracted raw data themes from the first four completed interviews utilizing an inductive or bottoms-up approach. The unit of analysis was any single coherent idea (unit of meaning), which included single words, phrases, sentences or paragraphs. It was possible to code a single unit for multiple raw data themes. The authors met after the initial round of manual, open coding, to compare and critically discuss their initial coding and to reach negotiated agreement where discrepancies and confusion existed. The authors collectively grouped the various codes into sub-categories. NK conducted, transcribed and analyzed the remaining 16 interviews with the previously developed coding scheme in a short space of time to increase coding stability. The authors met on multiple occasions during this period, critically discussing NK’s coding and categorization of the data and exploring alternative scenarios. The authors collectively grouped the sub-categories into categories and themes. Member checking and individual follow-ups ensured that the participant’s responses were correctly interpreted and to establish trustworthiness. The final results were made available to the participants and a feedback session was held with the coaching staff. The participants and coaches were asked to corroborate these findings or provide alternate views.

## Results

Tables 1–4 contain the main findings. Quotes were used to explain certain themes and categories. Table 1 contains five categories on the perceived psychological demands of elite rugby sevens. The players pointed to the tournament and match structure, extensive traveling, the high training load and the need to cope with the physicality of the sport as being psychologically very demanding.

**Table 1:** Perceptions about the psychological demands of elite rugby sevens.

Perceived psychological demands	Tournament structure	Ten tournaments on five continents	Long season
			Traveling for 2.5 months/year
		Match structure	Use every opportunity
			Mistake are costly
			Three matches per day
			Turnaround between matches
			Day 2 opponents: short notice
		Travel	Economy class flights
			Time zones/jet lag
			Fatigue, sleep difficulties
			Adapt to climates and cultures
	Resilience	Growth mind-set	Exposure to adversity
			Positive reaction to adversity
			Bounce back after setbacks
		Raise own standards	Comparison and competition with opposition and teammates
		Injuries	High-collision, high risk sport
			Inherent part of the game
			Physical rehabilitation
			Psycho-social recovery
	Cope with physicality	High training load	3–5 high intensity sessions/day
			Quality over quantity
		Push physical barriers	
		Play through niggles	
		Monitor recovery	Daily rating on phone app
	Perform when fatigued	Reduce negative effect	Peak conditioning
			Use various recovery strategies
		Maintain concentration	
	Perform under pressure	High expectations and performance standards	Self (internal competition)
			From others
		Consistency	Thrive on pressure

**Table 2:** Perceptions about the Blitzboks’ prevailing team culture.

Team culture	Team cohesion	Team comes before individuals (no superstars)	
		Brotherhood/family	
		Embrace cultural differences	
		Play for each other	
		Everybody on the “same page”	
	Clear purpose	Grow as people	
		Role-models to South African youth	
		Impact the lives of others	
	Work ethic	Team focused	Process driven
			Roles and responsibilities
		High work rate	
		Discipline	
		Feed of the energy of others	
	Team values	Respect, humility, loyalty, pride, passion, accountability, self-control, dedication, leadership	
	Happy environment	Relaxed environment	
		Enjoyment	
		Appreciative of each other	
	Relationship with coaching staff	Clear, open, honest communication	
		Aware of personality differences	
	Faith	God gave talents	
		Bible reading, prayer, listening to sermons	

**Table 3:** Perceptions about the use of psychological skills and mental strategies by elite rugby sevens players.

Psychological skills	Goal-directed behavior	Goal-setting	Clearly defined big picture
		Goal achievement	Strive for excellence
	Compartmentalization (When and how to switch on/off)	Focused attention (switch on)	Focus/refocus
			Routines
		Failure to switch off	Leads to overthinking
			Emotionally draining
			High burnout risk
	Deal with anxiety	Anxiety reduction	Lower anxiety levels
		Anxiety management	Interpret anxiety positively
	Motivation	Utilize various resources	Internal: drive, passion
			External: social support
	Imagery	Simulate matches	
		Visualize scenarios	
		Repetition/ rehearsal	
	Self-confidence	Meticulous preparation	Marginal gains (1%’s)
		Positive self-talk	Affirmation
		Previous performances	
Mental strategies	Coping	Emotional, relational, personal problems	Approach behavior
			Address personal issues
		Refereeing decisions, outside interference, setbacks, non-selection	Problem-focused coping
			Emotion-focused coping
	“Back-to-zero”	Restart after every play, break, match, day, result	
	Creativity	Big-box, small-box	Think outside the small box, still within the big box
			Cognitive triggers

**Table 4:** Well-being and individual needs of elite rugby sevens players.

Well-being and individual needs	Physical needs	Individualized training	Peak conditioning
			World-class facilities
			Weekly training plans
			Breaks in-between tournaments
		Individualized nutrition	Supplementation
			Hydration
			Top-up meals
		Access to expert medical team	Physicians, Physiotherapists, Biokineticists, massage therapy
	Financial concerns and needs	Earning less than fifteens	Code switching
		Earlier contracting	Security
			Stability
		Financial guidance	During and after career
	Preparation for life after rugby career	Shared responsibility between everyone	Players, agents, South African Rugby Union
		Furthering education	Certificates, coaching qualification
			More time to study
			Funding: Player or SA Rugby?
		Career guidance	Career termination exit strategy
			Networking
			Explore opportunities
			Start own business
		Holistic development (life – sport integration)	Develop sport identity in context
			Foster personal identity
	Support structures	Family/support system	Source of affirmation
			Explain team dynamics
			More family time
			Communication whilst traveling
		Mentorship needs	Mentor/life coach
			Act in the player’s best interest
	Mental (ill) health	Human/athlete condition	(Competitive) anxiety
		Subclinical mental health disorders	Insomnia
			Burnout
		Clinical mental health disorders	Depression
			Suicide ideation

The tournament structure of the international sevens series differs from fifteens:

“…six games over one weekend, you have six warm-ups. After every match you cool down, you’re a bit stiff. To pick yourself up after that first game, to do it all again the same day and go back and sleep. The next morning you feel like you ran in front of a train and you know there are three more games to come.” – P19

“If you are not mentally strong, you’re not going to make it here. A lot of the current Springboks [South African national fifteens side], said they will never come play sevens again, cause it’s just physically and psychologically too demanding.” – P1

Players have to use the available scoring opportunities and limit their own mistakes:

“The small decisions you have to make when you’re tired …There aren’t seconds in a sevens game; there are split seconds …You have to take every single opportunity the game throws at you. If you don’t, you can lose a game.” – P16

“…speed of the game. The room for error is tiny. One mistake and you probably lose a game. If you play a good team and have the ball, you must score.” – P12

Extensive economy class flights across multiple time zones pose challenges:

“…going from different time zones can be tough on the body, especially sleep wise. Luckily we get there a little in advance, we have two or three days to get our bodies ready …to adapt to the new surroundings.” – P17

Resilience is key to overcome injuries and other forms of adversity:

“I’ve been battling with injuries over the last two years. Struggling just to find my feet, getting back to playing good rugby and then getting injured. And I have to start from the floor again.” – P6

“Coping during physical training …Coping with disappointments, like injury and not making the team, stuff like that. You don’t only have to be tough for training, but also emotionally. Emotionally you have to be strong and tough, mentally tough, just to take everything as it comes.” – P10

Physical and mental fatigue constantly challenge the players:

“The recovery time between games is so short. You get a lot of bumps and bruises and you only have 2 h to recover. We do video analysis that is also draining mentally …you have to listen, your body is sore and you can lose focus quickly…it’s really hard on the body …there’s not a lot of time to recover before your next game …” – P14

Consistent performance necessitate that players develop across multiple domains:

“…I’ve dealt more with the mental side. I had two operations, so I’m definitely not physically at my best, but when I get my head right, I play well you know? I’m decisive and make smart decisions; the right ones at the right time.” – P12

Team culture was identified as a key contributor to the team’s performance, with seven categories outlined in Table 2.

A strong cohesiveness that embraced the rich diversity of the group was evident:

“…a family, we are really tight …we spend a lot time together, doing stuff together. Our team culture and dynamics is very important and really strong. We make sure that it does not go away regardless of what happens, win or lose, in good and bad times. The culture is big.” – P3

“Different players from different backgrounds all aiming for one thing was pretty special.” – P20

“To be understanding and respectful of different people’s cultures; we really put a big emphasis on that. As much as we respect individual cultures, the players need to respect the team culture and the team identity.” – P2

The team had clear objectives around personal growth and about being role-models:

“…we want to touch lives and continuously grow as people as well, not only as rugby players. When we strive toward those goals; touching people’s lives and putting smiles on other people’s faces, it really builds such a beautiful picture …it’s phenomenal really.” – P4

“Our motto is ‘touching people’s lives.’ Everywhere we go we try to change or make a good impression on people’s lives.” – P2

Work ethic is essential to the team culture:

“…these guys are grafters, pure work horses. It is probably the back bone of our setup …If you’re scared of work then sevens is not the game for you.” – P1

“…discipline is the most important thing. If you don’t have discipline you will just think of yourself and not care about the other guys around you. And the other thing is work ethic, you must work hard.” – P18

A number of the participants highlighted the role of faith in their lives and rugby careers:

“I’m a believer …so I think those challenges [referring to injuries] are preparing me for greater things that lie ahead. I read my Bible. My Bible is like, it’s not my stress reliever, but it calms me down and gives me hope that everything will be fine. That’s a very big motivation for me.” – P3

“…the talent is not yours; God gave you this talent. It depends what you do with it. You can touch other people’s lives or you can just play around with it.” – P18

“I don’t allow stress to affect me that much, because I’m a believer. I believe the Lord will never put you in a situation that you can’t handle. So, whenever I’m in a situation, I’m going to handle it, because I’m strengthened by the Lord.” – P20

Table 3 contains the psychological skills and mental strategies used by elite rugby sevens players.

Staying focused over a prolonged duration is a prerequisite for success, but some players noted that they need to deliberately shift their focus away from rugby:

“…mentally, you want to try and be at your best every single time. You need to know yourself first of all, and need to know the tools you can use …Being consistent is the most important …, before a game, you have your routine and you’re mentally ready every single time. Not just for games, also for training.” – P17

“…to focus on the right stuff. My mental challenge is to stop thinking about the game. Sometimes I overthink the game. I really play the game over in my mind a lot in terms of how we are going to play; if the defense does this then we need to do that, we need to do this, you know? I think uh, I sometimes burn myself out. …that is mentally draining, you know?” – P14

Different coping strategies are needed to deal with external factors:

“Emotional issues will always be there. To cut out all the baggage and leave it at home. So when you hit the field you have a clear and open mind to focus on your job, focus on your performance. Any emotional baggage that you carry is going to affect your performance on the field.” – P11

“Off-field or on-field, your mental state is probably most important. You can’t take personal stuff onto the field. …when you put your boots on next to the field, you switch on. You think about what’s going to happen on the field. You can’t let stuff that is bothering you at home, family, anything, mix with your rugby …You need to be in a good mental state to play the game.” – P15

Sustained performance at this elite level requires a strong inner drive:

“It’s a very demanding sport in terms of mental preparation and mental training, but if you’re not up for the challenge from the get go and [don’t have] that burning desire to want it more than the other guys, then you’re not going to make it.” – P1

“…you need to motivate yourself, even though it is a team environment. You need to do what you do right, be the piece of the puzzle for the team.” – P3

Imagery assists in preparing for various on-field scenarios:

“…a product of how many times you see things on the field. If you are in a situation and you haven’t seen it before, you start to get very nervous, cause you don’t know how to handle it or how you’re going to react to the situation. But if you’ve seen a situation a couple times before, you can be a lot calmer.” – P12

The team employ a “big-box, small-box” philosophy referring to the creativity players are encouraged to produce within the bigger game plan:

“…everyone’s got this blueprint that we’ve been given for a tournament and within that blueprint we’ve got so much freedom to let your natural abilities come through, and to express yourself.” – P4

Whilst peak performance is paramount at the elite level there is a growing responsibility to address player well-being and their off-the-field needs. Table 4 contains four categories relating to the current and future well-being needs.

Most of the players acknowledged the importance of adequate financial planning and furthering their own education in preparation of the inevitable career change from professional rugby to a post-rugby career. Despite not mentioned frequently, the need to address various mental health issues and fostering general well-being is a high priority. A number of players require greater financial security and financial guidance:

“…sevens players are not getting paid as much as the fifteens guys …What would make it better is to match what the fifteens guys are getting …, so that money doesn’t force a player to go to fifteens. I would’ve loved to have made enough money and to have found something that I want to do after rugby, because right now I have no clue. I’m still trying to figure it out. I would have loved to have things in place that’s going to sustain me for the rest of my life.” – P20

“…my concern is that 10 years down the line one of my teammates will be like ‘Hey, do you have a job for me?’ There’s a lot of guys that come from nothing and unfortunately will go back to nothing, because [their] money is squandered. Then the opportunity for education has passed and you can’t retire on a sevens salary. You should be smart and put yourself in a good position.” – P12

The players are also aware that they need to start preparing early for life after rugby:

“A lot of guys don’t study after school. But some of the guys do and it can add a lot of pressure, you know? Playing rugby and also study at the same time, so you’re like a student-athlete sorta thing …, you need to have a backup plan …” – PP17

Mentorship within the team seems to have a positive effect of well-being:

“…there are very good leaders, because when I came here, they took me under their wing and they actually made something of me.” – P7

Success on the world stage doesn’t safeguard athletes from mental health challenges:

“I have these heavy anxiety feelings about …what if I got injured? The anxiety and fear literally want to lock you up and make you sit down in a dark corner. Just leave me please, don’t let me play, I don’t want to play. And there has been a lot of times in my career when I’ve been like uh, you know, pick somebody else. Like I don’t want to be picked, you know? Because what if you mess up? …it gets hectic sometimes. It goes far deeper. Sometimes you even sit there and you’re like ‘Well, what if I wasn’t even here? Then I wouldn’t have to play rugby. Do you know what I mean when I say I’m not here? Like dead”’. – P12

## Discussion

### Psychological Demands

The current results broaden our understanding of the demands of elite rugby sevens, the way in which this format of the game differs from fifteens and possible reasons why fifteens players may struggle with the transition to sevens. The tournament structure and schedule, multiple matches, short recovery periods and extensive traveling across multiple time zones is taxing and contribute to the experience and accumulation of both physical and mental fatigue (Waterhouse et al., 2000). Economy class flights caused further physical discomfort. To reduce the adverse effect of traveling, Meir (2012) suggest that players stay hydrated during flights, adapt their sleeping patterns and display a positive attitude. Set training routines and sleeping times could also minimize the negative after-effects (Waterhouse et al., 2000). Despite such efforts, sleep quality and quantity is often low (Fullagar et al., 2015). Training methods should focus on recovery to reduce injuries and fatigue (Higham et al., 2012, 2013; Lopez et al., 2012; Meir, 2012). Due to the short duration of matches, a single error could cost you a match or tournament (Meir, 2012) and players have to use every scoring and attacking opportunity (Barkell et al., 2016).

Toohey et al. (2019) reported an injury incident rate of 45.0 injuries per 1000 player-hours among 55 elite Australian male rugby sevens players over a 2-year period, with a high rate of subsequent injuries. The collisional nature of the sport coupled with fatigue taxes players physically, psychologically and socially. The high risk of injury and re-injury necessitates that players develop mental toughness and resilience to overcome these and other adverse stressors. Adverse experiences (e.g., non-selection, significant sporting failure, injuries, personal life challenges) plays a vital role in the psychological and performance development of elite athletes as it could result in adaptive behavioral responses and increased resilience (Sarkar et al., 2015). Sarkar and Fletcher (2017) continued that adversity-related experiences (including non-sport life experiences) combined with growth-related processes are essential for success at the elite level. The team’s growth mind-set bodes well for turning possible adverse situations into learning opportunities. High performance expectations and standards are an innate part of elite sport. The current results point to multiple sources of pressure to perform consistently (e.g., self-imposed, team members, external pressure). Henriksen et al. (in press) noted that high performance demands coupled with high training loads places athletes at increased risk of developing mental health problems, requiring that psychological demands such as the ones identified in the current study be addressed to enhance mental health and well-being. In fact, they propose that mental health should be a core component of a culture of excellence.

### Team Culture

The importance of strong team cohesion is well documented (see Carron et al., 2000; Kozub and Button, 2000). A positive team environment fosters strong team cohesion (McLaren et al., 2017), as the players play for each other rather than focusing on their personal accomplishments. The team’s strong cohesiveness could also be due to their success, because of the reciprocal cohesion-performance relationship (Asamoah and Grobbelaar, 2017). The group embraced their diversity (e.g., different backgrounds, cultures, personalities), challenging conventional thinking that similarity among group members is necessary for strong cohesion. These differences appeared trivial in comparison to the squad members having the same work ethic (deemed crucial to the team’s success) and an extensive list of shared team values. Sport is renowned as a vehicle for social change and empowerment, and plays an important role in the South African context, due to the country’s history of racial and class segregation (Merrett, 2006). Embracing each other’s differences and uniqueness is a distinctive feature of the team that contribute to their purpose of being role models who inspire the youth and influence the lives of all South Africans. Personal qualities, a facilitative environment and a challenge mind-set are prerequisites to develop psychological resilience for sustained success (Fletcher and Sarkar, 2016). The data showed that all three of these conditions were evident, potentially contributing to the team’s successful defense of the 2016/2017 World Series title in 2017/2018.

The team culture is characterized by healthy player-coach relationships and creating an environment in which players can enjoy what they are doing, to the extent that smiles on the player’s faces are indicative of an effective system. On the individual level, faith and religious beliefs also affected the team culture, whilst being cognizant of individual differences. Prayer has been noted to aid team cohesion (Murray et al., 2005), as it enhances a sense of unity among teammates (Turman, 2003). Many athletes use prayer and Bible reading as specific coping strategies (Park, 2000; Egli et al., 2014; Osborne Finekaso and Treharne, 2019).

### Psychological Skills and Mental Strategies

The study did not yield any groundbreaking information, but pointed to an extensive use of a wide variety of psychological skills and mental strategies by these players in response to the perceived psychological and performance demands. Whereas the available resources allow for the service of a sport psychologist for the national team, the need exist to develop and implement a comprehensive sport psychological skills development program for players from grassroots level. Players should be exposed to skills such as self-talk, imagery and attentional focus to develop mental toughness from a young age (Di Corrado et al., 2014). A combination of psychological skills seems effective in lowering or managing competition anxiety (Neil et al., 2006; Sharp et al., 2013). Cognitive-general imagery (visualizing scenarios and rehearsing strategies) helps to prepare for upcoming matches as the next opponent is usually finalized around 2 h before kick-off. Disengaging from relational and personal issues whilst training and competing had a marked effect on attention. Likewise, they switch their attention away from the sport during off-periods, as failure to do so could increase chronic stress; a key contributor to burnout (Raedeke, 1997).

Individual coping mechanisms was utilized in response to personal and relational problems (emotional-focused coping), disappointments, setbacks, injuries, non-selection, handling negative feedback (problem and/or emotion-focused coping). Osborne Finekaso and Treharne (2019) observed a preference among Fijian sevens rugby players to deploy emotion-focused coping strategies to reduce stress.

### Well-Being and Individual Needs

The players received limited financial and career guidance. Earlier contacting would also enable them to plan better (Stambulova et al., 2009). Whilst it is not clear who should pay for further education, there is consensus that players should be encouraged to study further. Failure to plan ahead and not having post-career financial and lifestyle provisions in order may exacerbate the transitional distress many players experience after terminating their playing careers (Henriksen et al., in press). A lack of work experience is often a major concern as retirement becomes eminent (Wylleman and Reints, 2010), and players should be granted opportunities to experience other workplace environments and to establish networks.

The effort needed to achieve success at the elite level restricts opportunities for identity development beyond the athletic role (Creswell and Eklund, 2005), and many athletes struggle to balance their personal lives and careers (Stambulova et al., 2009). The development of a positive sporting identity is an important resource for a player at the peak of their playing career, but may become a barrier once they retire from elite sport (Hickey and Kelly, 2008). Personal development should be prioritized (Martindale and Mortimer, 2011) to foster personal and sport identity from the time a player starts to specialize in a specific sport (Wylleman et al., 2004). Support networks and family relationships should be nurtured as it influences athlete well-being (Sharp et al., 2013).

The player who discussed his ongoing challenge with depression and suicide ideation (and gave consent to write about it) should be commended as Henriksen et al. (in press), noted that such actions may promote others to open up about their mental health challenges, thereby normalizing these experiences and potentially reducing the stigmas attached to mental ill health. Identifying an elite player who experience common mental health disorder symptoms is not surprising, as elite athletes and non-athletes have similar prevalence rates. In fact, injuries, overtraining and career termination could increase the risk of athletes developing mental health disorders (Moesch et al., 2018). Mental health disorder symptoms are more prevalent among athletes who lack social support, suffered severe injuries or experienced difficult life events (Schinke et al., 2017). Gouttebarge et al. (2018) observed a high 12-month prevalence of common mental health disorder symptoms among 595 male professional rugby players: distress (11%), eating disorders (11%), sleep disturbance (12%), adverse alcohol use (22%), and anxiety/depression (28%). Ninety-five percent of the participants noted a detrimental effect on performance, whilst 46% noted that current rugby structures were inadequate to address mental health. Rugby governing bodies should increase awareness about common mental health disorders among current and retired players, and put preventive and support measures in place (Gouttebarge et al., 2016).

Leburn and Collins (2017) alluded to the possible ineffectiveness of current clinical screening methods, due to the additional performance stressors elite athletes’ experience. The consensus statement on improving mental health of high performance athletes (Henriksen et al., in press), warns against pathologizing normal human experiences. They expressed the need to distinguish between clinical mental health disorders, subclinical mental ill health, the human condition (i.e., periodic and completely normal experiences of adversity, unpleasant thoughts and emotions in response to general life) and the athlete condition (e.g., performance anxiety in response to competition).

## Conclusion and Recommendations

The thick descriptive information pertaining to the perceived psychological demands of the game, the prevailing team culture, the implementation of psychological skills and mental strategies, as well as the well-being needs of elite players identified through this study could assist rugby governing bodies to support and attend to their player’s needs more effectively. These findings should be integrated with evidence-based best practice regarding sport psychological skills and well-being development within a holistic framework aimed at improving performance as well as fostering well-being during and after retirement from elite sport. The effectiveness of such a program should be tested empirically.

## Author Contributions

NK explored the perceived demands of rugby sevens held by professional players and coaching staff, conducted, transcribed and coded all the semi-structured interviews, and wrote up the results and discussion as part of the original M.Sc. thesis. HG initially conceptualized the research problem, study design and methodology, contributed to the qualitative analysis, wrote the article from the thesis, and submitted the article.

## Conflict of Interest Statement

The authors declare that the research was conducted in the absence of any commercial or financial relationships that could be construed as a potential conflict of interest.

## References

[B1] Asamoah B. Grobbelaar H. W. (2017). Team cohesion and performance during a university soccer championship: two sides of the coin. *SA J. Res. Sport Phys. Educ. Recr.* 39 1–15.

[B2] Barkell J. F. O’Connor D. Cotton W. G. (2016). Characteristics of winning men’s and women’s sevens rugby teams throughout the knockout cup stages of international tournaments. *Int. J. Perf. Anal. Sport* 16 633–651. 10.1080/24748668.2016.11868914

[B3] Braun V. Clarke V. (2006). Using thematic analysis in psychology. *Qual. Res. Psychol.* 3 77–101. 10.1191/1478088706qp063oa

[B4] Carreras D. Kraak W. Planas A. Martin I. Vaz L. (2013). Analysis of International rugby sevens matches during tournaments. *Int. J. Perf. Anal. Sport* 13 833–847. 10.1080/24748668.2013.11868692

[B5] Carron A. V. Colman M. M. Wheeler J. Stevens D. (2000). Cohesion and performance in sport: a meta-analysis. *J. Sport Exerc. Psychol.* 24 168–188. 10.1123/jsep.24.2.168

[B6] Creswell S. L. Eklund R. C. (2005). Changes in athlete burnout and motivation over a 12-week league tournament. *Med. Sci. Sport Exerc.* 37 1957–1966. 10.1249/01.mss.0000176304.14675.32 16286867

[B7] Di Corrado D. Murgia M. Freda A. (2014). Attentional focus and mental skills in senior and junior professional rugby union players. *Sport Sci. Health* 10 79–83. 10.1007/s11332-014-0177-x

[B8] Egli T. Czech D. R. Todd S. Y. Shaver G. W. Gentner N. Biber D. D. (2014). The experience of Christian prayer in coaching: a qualitative investigation. *J. Psychol. Christ.* 33 45–57.

[B9] Fletcher D. Sarkar M. (2016). Mental fortitude training: an evidence-based approach to developing psychological resilience for sustained success. *J. Sport Psychol. Action* 7 135–157. 10.1080/21520704.2016.1255496

[B10] Fullagar H. H. K. Duffield R. Skorski S. Coutts A. J. Julian R. Meyer T. (2015). Sleep and recovery in team sport: current sleep-related issues facing professional team-sport athletes. *Int. J. Sport Physiol.* 10 950–957. 10.1123/ijspp.2014-0565 25756787

[B11] Gouttebarge V. Hopley P. Kerkhoffs G. Verhagen E. Viljoen W. Wylleman P. (2018). A 12-month prospective cohort study of symptoms of common mental disorders among professional rugby players. *Eur. J. Sport Sci.* 25 1–9. 10.1080/17461391.2018.1466914 29698129

[B12] Gouttebarge V. Kerkhoffs G. Lambert M. (2016). Prevalence and determinants of symptoms of common mental disorders in retired professional rugby union players. *Eur. J. Sport Sci.* 16 595–602. 10.1080/17461391.2015.1086819 26419657

[B13] Hendricks S. (2012). Trainability of junior rugby union players. *S. Afr J. Sports Med.* 24 122–126. 10.17159/2078-516X/2012/v24i4a525

[B14] Hiatt J. F. (1986). Spirituality, medicine, and healing. *S. Med. J.* 79 736–743. 10.1097/00007611-198606000-00022 3715539

[B15] Hickey C. Kelly P. (2008). Preparing to not be a footballer: higher education and professional sport. *Sport Educ. Soc.* 13 477–494. 10.1080/13573320802445132

[B16] Higham D. G. Pyne D. B. Anson J. M. Eddy A. (2012). Movement patterns in rugby sevens: effects of tournament level, fatigue and substitute players. *J. Sci. Med. Sport* 15 277–282. 10.1016/j.jsams.2011.11.256 22188846

[B17] Higham D. G. Pyne D. B. Anson J. M. Eddy A. (2013). Physiological, anthropometric and performance characteristics of rugby sevens players. *Int. J. Sport Physiol.* 8 19–27. 10.1123/ijspp.8.1.19 22868376

[B18] Kozub S. A. Button C. J. (2000). The influence of a competitive outcome on perceptions of cohesion in rugby and swimming teams. *Int. J. Sport Psychol.* 31 82–95.

[B19] Leburn F. Collins D. (2017). Is elite sport (really) bad for you? Can we answer the question? *Front. Psychol.* 8:324. 10.3389/fpsyg.2017.00324 28316585 PMC5334321

[B20] Lopez V. Galano G. J. Black C. M. Gupta A. T. James D. E. Kelleher K. M. (2012). Profile of an American amateur rugby union sevens series. *Am. J. Sport Med.* 40 179–184. 10.1177/0363546511427124 22102102

[B21] MacNamara A. Button A. Collins D. (2010a). The role of psychological characteristics in facilitating the pathway to elite performance. Part 1: identifying mental skills and behaviours. *Sport Psychol.* 24 52–73. 10.1123/tsp.24.1.52

[B22] MacNamara A. Button A. Collins D. (2010b). The role of psychological characteristics in facilitating the pathway to elite performance. Part 2: examining environmental and stage-related differences in skills and behaviors. *Sport Psychol.* 24 74–96. 10.1123/tsp.24.1.74

[B23] Martindale R. Mortimer P. (2011). “Talent development environments: key considerations foe effective practice,” in *Performance Psychology: A Practitioner’s Guide*, eds Collins D. Button A. Richards H. (London: Elsevier), 65–84.

[B24] McLaren C. D. Newland A. Eys M. Newton M. (2017). Peer-initiated motivational climate and group cohesion in youth sport. *J. Appl. Psychol.* 29 88–100. 10.1080/10413200.2016.1190423

[B25] Meir R. A. (2012). Training for and competing in sevens rugby: practical considerations from experience in the international rugby board world series. *Strength Cond. J.* 34 76–86. 10.1519/SSC.0b013e31825105ed

[B26] Merrett C. (2006). Sport, segregation and space: the historical geography of physical recreation in the South Africa. *Hist. Compass.* 5 1–10. 10.1111/j.1478-0542.2006.00368.x

[B27] Moesch K. Kenttä G. Kleinert J. Quignon-Fleuret C. Cecil S. Bertollo M. (2018). FEPSAC position statement: mental health disorders in elite athletes and models of service provision. *Psychol. Sport Exerc.* 38 61–71. 10.1016/j.psychsport.2018.05.013

[B28] Murray M. A. Joyner A. B. Burke K. L. Wilson M. J. Zwald A. D. (2005). The relationship between prayer and team cohesion in collegiate softball teams. *J. Psychol. Christ.* 24 233–239.

[B29] Neil R. Mellalieu D. S. Hanton S. (2006). Psychological skills usage and the competitive anxiety response as a function of a skill level in rugby union. *J. Sport Sci. Med.* 5 415–423. 24353459 PMC3842142

[B30] Osborne Finekaso G. Treharne G. J. (2019). Stress and coping in Fijian rakavi (rugby) sevens players. *Sport Soc.* (in press). 10.1080/17430437.2018.1487954

[B31] Park J. (2000). Coping strategies used by Korean national athletes. *Sport Psychol.* 14 63–80. 10.1123/tsp.14.1.63

[B32] Raedeke T. (1997). Is athlete burnout more than just stress? A sport commitment perspective. *J. Sport Exerc. Psychol.* 19 396–417. 10.1123/jsep.19.4.396

[B33] Sarkar M. Fletcher D. (2017). Adversity-related experiences are essential for Olympic success: additional evidence and considerations. *Prog. Brain Res.* 232 159–165. 10.1016/bs.pbr.2016.11.009 28648237

[B34] Sarkar M. Fletcher D. Brown D. J. (2015). What doesn’t kill me: adversity-related experiences are vital in the development of superior Olympic performance. *J. Sci. Med. Sport* 18 475–479. 10.1016/j.jsams.2014.06.010 25035123

[B35] Schinke R. J. Stambulova N. B. Si G. Moore Z. (2017). International society of sport psychology position stand: athletes’ mental health, performance, and development. *Int. J. Sport Exerc. Psychol.* 2 1–18. 9239993

[B36] Sharp L. A. Woodcock C. Holland M. J. G. Coming J. Duda J. L. (2013). A qualitative evaluation of the effectiveness of a mental skills training program for youth athletes. *Sport Psychol.* 27 219–232. 10.1123/tsp.27.3.219

[B37] Stambulova N. Alfermann D. Statler T. Côté J. (2009). ISSP position stand: career development and transitions of athletes. *Int. J. Sport Exerc. Psychol.* 7 395–412. 10.1080/1612197X.2009.9671916

[B38] Suarez-Arrones L. J. Nuñez F. J. Portillo J. Mendez-Villanueva A. (2012). Running demands and heart rate responses in men rugby sevens. *J. Strength Cond. Res.* 26 3155–3159. 10.1519/JSC.0b013e318243fff7 22158098

[B39] Taylor J. (1995). A conceptual model for integrating athletes’ needs sport demands in the development of competitive mental preparation strategie.s *Sport Psychol.* 9 339–357. 10.1123/tsp.9.3.339

[B40] Toohey L. A. Drew M. K. Finch C. F. Cook J. L. Fortington L. V. (2019). A 2-year prospective study of injury epidemiology in elite Australian rugby sevens: exploration of incidence rates, severity, injury type, and subsequent injury in Men and Women. *Am. J. Sports Med.* 10.1177/0363546518825380 [Epub ahead of print]. 30779880

[B41] Tucker R. (2016). Rugby sevens: Olympic debutante and research catalyst. *Br. J. Sport Med.* 50 638–639. 10.1136/bjsports-2016-096306 27190228

[B42] Turman P. D. (2003). Coaches and cohesion: the impact of coaching techniques on team cohesion in the small group sport setting. *J. Sport Behav.* 26 86–104.

[B43] Van Rooyen M. (2015). Early success is key to winning an IRB sevens world series. *Int. J. Sports Sci. Coach.* 10 1129–1138. 10.1260/1747-9541.10.6.1129

[B44] Waterhouse J. Reilly T. Atkinson G. (2000). Chronobiological consequences of long haul flights, travel fatigue, and jet lag. *Int. Sportmed J.* 1 1–9.

[B45] World Rugby (2016). *HSBC Sevens World Series Website.* Available at: https://www.world.rugby/sevens-series (accessed April 6 2016).

[B46] Wylleman P. Alfermann D. Lavallee D. (2004). Career transitions in sport: European perspectives. *Psychol. Sport Exerc.* 5 7–20. 10.1016/S1469-0292(02)00049-3

[B47] Wylleman P. Reints A. (2010). A lifespan perspective on the career of talented and elite athletes: perspectives on high-intensity sports. *Scand. J. Med. Sci. Sport* 20 88–94. 10.1111/j.1600-0838.2010.01194.x 20840566

